# Analysis of the Effect of Fabrication Parameters on the Properties of Biopolymer Coatings Deposited on Ti13Zr13Nb Alloy

**DOI:** 10.3390/polym17233136

**Published:** 2025-11-25

**Authors:** Michał Bartmański, Kamila Sionek

**Affiliations:** Department of Biomaterials Technology, Faculty of Mechanical Engineering and Ship Technology, Gdańsk University of Technology, 82-233 Gdańsk, Poland

**Keywords:** chitosan-based coatings, electrophoretic deposition (EPD), Ti13Zr13Nb alloy, nanoparticles, corrosion resistance, biocompatibility

## Abstract

This work describes the preparation and characterization of chitosan-based biopolymer coatings containing silver, zinc, and hydroxyapatite nanoparticles deposited on the Ti13Zr13Nb alloy by the EPD method. It was intended to evaluate the influence of surface pretreatments and deposition parameters on the structural, electrochemical, and biological properties of coatings. The morphology and composition were characterized by means of SEM/EDS, AFM, XRD, and FTIR analysis. The obtained results indicated uniform continuous layers with homogeneously distributed nanoparticles and the presence of characteristic functional groups originating from chitosan and hydroxyapatite. Corrosion investigations performed in SBF solution revealed a significant enhancement in corrosion resistance for chitosan/nanoAg/nanoZn/nanoHAp coatings, reflected in a drastic decrease in corrosion current density compared with uncoated Ti13Zr13Nb alloy. The contact angle measurements confirmed their hydrophilic nature, which favors better biointegration ability. Biological tests (MTT and LDH) performed on human osteoblasts (hFOB 1.19) confirmed high biocompatibility (>85% cell viability) in the case of all coatings with the addition of hydroxyapatite, whereas in the case of coatings without HAp, cytotoxicity was observed, probably due to the uncontrolled release of metallic nanoparticles. These findings suggest that the presence of hydroxyapatite in chitosan-based coatings efficiently enhances corrosion protection and cytocompatibility, showing very good prospects for biomedical applications such as the surface modification of titanium implants.

## 1. Introduction

Chitosan belongs to the polymers most often applied to deposit coatings for several medical and biological applications, in particular for titanium implants [1,2]. It can be used alone as a coating, but because of its weak mechanical properties and also the need to enhance the biological behavior, some metallic nanoparticles become the second component of the coatings. Among them, silver and zinc, in elementary or chemical compound forms, are the most popular. The deposition techniques of chitosan-based coatings are the electrophoretic deposition (EPD), layer-by-layer (LBL) method, dip and spin coating, and solution casting [1]. Enrichment of chitosan with nanosilver was quite often found in medical applications, in different forms and medical domains. The most commonly applied are chitosan-Ag coatings for antibacterial protection. The two-component chitosan-Ag coatings were shown to be effective for such a purpose after EPD formation on Ni-Ti alloy [3,4]. The chitosan film decorated with Ag NPs [5] demonstrated antibacterial efficacy against the other pathogens, the Gram-positive *B. subtilis* and Gram-negative *E. coli* bacteria cells. More complex material solution included PVA-capped AgNPs in chitosan matrix with bactericidal activity against *E. coli* and *S. aureus* [6], and as Ag@ZnO films on chitosan/PVA, similar effects regarding the same strains [7]. In addition, the chitosan-coated silver nanoparticles were proposed against SARS-CoV-2 [8]. As concerns these coatings in fighting cancer cells, in [9] the chitosan-coated Ag NPs were synthesized using Moringa oleifera flower extract and used against triple-negative breast cancer, in [10] as sericin-chitosan implemented with AgNPs against colon cancer [11]. Further, the PVA/chit biofilms doped with tea tree oil and enhanced with AgNPs were investigated in dressing wounds can be used [12]. Finally, chitosan with nanoAg was repeatedly shown to be highly effective in food preservation against bacteria and fungi [13,14,15,16,17]. In chitosan–zinc coatings, zinc is usually applied in its oxide form [18,19]. In the simplest form, the chitosan coating with added ZnONPs deposited on nanotubular TiO_2_ showed the antibacterial activity against *E. coli* strain [19]. In [20] chitosan–calcium phosphate double layer with embedded ZnO and amikacin, created by micro-arc oxidation technique, was observed to be significantly effective against a mixture of various hospital bacteria, including MRSA (methicillin-resistant Staphylococcus aureus). For other composite coatings [21] containing chitosan, molybdenum disulfide, and ZnONPs showed antibacterial efficiency versus *S. aureus* and *E. coli*. Similarly to nanosilver, ZnO was also applied for food preservation, such as [22] the garlic extract—zinc oxide NPs—chitosan—PVA for fish food, chitosan—ZnONPs for lemon pomace [23], in [24] together with AgNPs for sea products, and also in [25] as Ti/ZnO/SiOx/chitosan coatings for food preservation.

## 2. Materials and Methods

### 2.1. Substrate Material Preparation

The Ti13Zr13Nb alloy (SeaBird Metal Materials Co., Baoji, China) used as the substrate had the chemical composition shown in Table 1. Disc-shaped specimens, 4 mm in thickness, were sectioned from rods with a diameter of 20 mm. The surfaces were mechanically ground using a series of abrasive papers, with grit no. 2000 as the final step, resulting in an average surface roughness of Sa = 0.13 µm. Subsequently, the specimens were ultrasonically cleaned (Sonic-3, POL-SONIC, Warsaw, Poland) for 15 min in pure isopropanol (Polskie Odczynniki Chemiczne, POCH, Gliwice, Poland), followed by rinsing with deionized water.

### 2.2. Electrochemical Oxidation of Ti13Zr13Nb Alloy

Electrochemical oxidation was carried out in an electrolyte composed of 10 mL of 85% orthophosphoric acid (1 M H_3_PO_4_; Sigma-Aldrich, St. Louis, MO, USA), 1.2 mL of 40% hydrofluoric acid (HF; Polskie Odczynniki Chemiczne, POCH, Gliwice, Poland), and 150 mL of deionized water. The process was performed using a standard electrochemical setup consisting of a cell connected to a DC power supply (MCP/SPN110-01C, Shanghai MCP Corp., Shanghai, China), with a platinum electrode serving as the cathode and the Ti13Zr13Nb alloy specimen as the anode, positioned at a distance of 10 mm. Oxidation was conducted at room temperature under a constant voltage of 20 V for 20 min. Following the treatment, the specimens were thoroughly rinsed with distilled water and air-dried at ambient temperature for 24 h.

### 2.3. Electrophoretic Deposition of Coatings

The electrolytes were prepared by dispersing 1 g of high-molecular-weight chitosan (degree of deacetylation > 75%; mol wt. 310,000–375,000 Da; Coarse ground flakes and powder form; CAS Number: 9012-76-4; Sigma-Aldrich, St. Louis, MO, USA) in 1 L of 1% (*v*/*v*) acetic acid (Polskie Odczynniki Chemiczne, POCH, Gliwice, Poland). The solutions were homogenized using a magnetic stirrer at 250 rpm for 24 h at room temperature. One hour prior to electrophoretic deposition (EPD), 0.05 g/L of silver nanopowder (Hongwu International Group Ltd., Guangzhou, China) with an average particle size of approximately 30 nm, 0.05 g/L of zinc nanopowder (MKNano, Mississauga, Canada) with an average particle size of approximately 50 nm, and 2.5 g/L of hydroxyapatite nanopowder (MKNano, Mississauga, Canada) with an average particle size of approximately 20 nm were added to the chitosan electrolyte. Additionally, 10 mL/L of Tween 20 (Sigma-Aldrich, St. Louis, MO, USA) was introduced, and the suspension was homogenized in an ultrasonic bath for 1 h at room temperature. The Ti13Zr13Nb specimens with TiO_2_ layers served as the cathode, while a platinum plate was used as the anode, with an electrode spacing of 10 mm. The deposition was performed using a DC power supply (MCP/SPN110-01C, Shanghai MCP Corp., Shanghai, China) at applied voltages of 20 V for 1 at room temperature. After deposition, the composite coatings were rinsed with distilled water and air-dried at ambient temperature for 48 h. Details of the electrophoretic deposition parameters are presented in Table 2.

### 2.4. Characterization of Microstructure

The Ti13Zr13Nb griding alloy, nanotubular TiO_2_ layer and coating surface were examined for each specimen with a high-resolution scanning electron microscopes (SEM JEOL JSM-7800 F, JEOL Ltd., Tokyo, Japan) equipped with a LED detector at 5 kV or 15 kV acceleration voltage.

The chemical composition was determined using energy-dispersive X-ray spectroscopy (EDS) with a detector integrated into the scanning electron microscope (SEM).

The surface topography was examined using an atomic force microscope (NaniteAFM, Nanosurf AG, Liestal, Switzerland). The measurements were carried out in non-contact mode at a setpoint of 20 nN. The surface roughness parameter (Sa) was determined as the average value from three measurements performed over an area of 50 μm × 50 μm.

Phase identification was carried out using X-ray diffraction (XRD; Philips X’Pert Pro, Eindhoven, The Netherlands) with a diffractometer equipped with a Cu Kα radiation source (λ = 0.1554 nm), over a 2θ range of 10–90°, with a step size of 0.02° and a counting time of 17.34 s per point, under ambient temperature and atmospheric pressure conditions.

Fourier-transform infrared (FTIR) spectra were recorded using a spectrophotometer (Perkin Elmer Frontier, Poznań, Poland) at a resolution of 2 cm^−1^ within the range of 400–4000 cm^−1^.

The thickness of the obtained coatings was measured using an Isoscope-type coating thickness gauge (FMP10-20, Helmut Fischer GmbH, Sindelfingen, Germany).

### 2.5. Corrosion Studies

Corrosion tests were carried out using a potentiostat (Atlas 0531, Atlas-Sollich, Gdańsk, Poland) and AtlasCorr05 software. A three-electrode system was applied, in which the sample served as the working electrode, a platinum wire acted as the counter electrode, and a saturated calomel electrode (SCE) was used as the reference electrode. During the corrosion measurements, all electrodes were immersed in artificial saliva (SBF) prepared according to the composition described below. The SBF solution contained the following components: urea ((NH_2_)_2_CO, ACROS Organics, 0.39 g/L), sodium chloride (NaCl, CHEMPUR, 2.10 g/L), sodium bicarbonate (NaHCO_3_, CHEMPUR, 4.50 g/L), disodium hydrogen phosphate (Na_2_HPO_4_, CHEMPUR, 0.78 g/L), dipotassium hydrogen phosphate (K_2_HPO_4_, ACROS Organics, 0.60 g/L), potassium thiocyanate (KSCN, ACROS Organics, 0.99 g/L), and potassium chloride (KCl, CHEMPUR, 3.60 g/L). A magnetic stirrer was used to maintain a constant temperature of 37 °C. The open-circuit potential (OCP) was recorded prior to polarization measurements. The corrosion resistance was evaluated by potentiodynamic polarization within a potential range from −1.0 V to +1.0 V at a scan rate of 1 mV/s. The initial and final potentials were held for 10 s each. The corrosion potential (E_corr) and corrosion current density (i_corr) were determined by extrapolating the Tafel slopes using the built-in AtlasLab software (version 9, Atlas-Sollich, Rębiechowo, Poland).

### 2.6. Contact Angle Studies

The water contact angle was measured using the sessile drop method with a goniometer (Attension Theta Lite, Biolin Scientific, Espoo, Finland) at room temperature. Four measurements were performed for each sample. The volume of the distilled water droplets was approximately 2 μL, and the contact angle was recorded for 10 s after the droplet was deposited on the surface.

### 2.7. Evaluation of the Biological Properties of the Obtained Coatings

The cellular response was evaluated using a human osteoblast cell line hFOB 1.19 (RRID: CVCL_3708) obtained from the American Type Culture Collection (ATCC). The cells were cultured in a 1:1 mixture of Ham’s F12 medium and Dulbecco’s Modified Eagle Medium (DMEM, without phenol red) supplemented with 0.3 mg/mL G418 and 10% fetal bovine serum (FBS) at 37 °C. To assess the indirect effect of the tested samples on cellular response, the cells were treated with conditioned medium obtained by incubating the samples under identical conditions as those used for cell culture. The cells were seeded at a density of 0.012 × 10^6^ cells per well in 96-well culture plates and cultured under standard conditions for 24 h. The medium was then replaced with the extract obtained from the tested material, and the culture was continued for an additional 48 h. Cell viability after exposure to the tested materials was determined using the MTT reduction assay. The MTT test is a sensitive and reliable indicator of cellular metabolic activity. It is based on the reduction of the yellow, water-soluble tetrazolium salt 3-(4,5-dimethylthiazol-2-yl)-2,5-diphenyltetrazolium bromide (MTT) by mitochondrial dehydrogenases into insoluble purple formazan crystals. After incubation, the resulting formazan crystals were dissolved, and the absorbance of the solution was measured spectrophotometrically. The absorbance intensity is directly proportional to the number of viable cells. After 48 h of incubation in the conditioned medium, the culture medium was replaced with fresh medium containing 0.06 mol/L MTT, and the cells were incubated for an additional 4 h to allow for MTT metabolism by viable cells. The formazan crystals formed were then dissolved in 10% SDS solution, and the absorbance of the resulting product was measured using a spectrophotometer at a wavelength of 570 nm with a reference wavelength of 690 nm. Cells cultured in extracts from the unmodified Ti13Zr13Nb alloy served as the control group. Cell mortality was assessed using the lactate dehydrogenase (LDH, EC 1.1.1.27) release assay. LDH is a cytoplasmic enzyme released by dead or damaged cells. After 48 h of cell incubation in the extracts, the culture medium was collected, and LDH activity was measured. The oxidation of NADH was determined spectrophotometrically at a wavelength of 340 nm. The positive control in the assay consisted of 100% dead cells cultured in the extract from the unmodified Ti13Zr13Nb titanium alloy. Cell death was induced by adding Triton X-100 (final concentration 0.1%; Merck, Darmstadt, Germany) to the culture medium.

### 2.8. Statistic

The results are presented as means ± standard deviations (SD), and the obtained relationships were evaluated using one-way ANOVA followed by Tukey’s True Significant Difference post hoc test, with statistical significance set at *p* < 0.05. The normality of the data distribution was verified using the Shapiro–Wilk test. All statistical analyses were performed using GraphPad Prism 10 (GraphPad Software, Boston, MA, USA).

## 3. Results and Discussion

**Figure 1:**
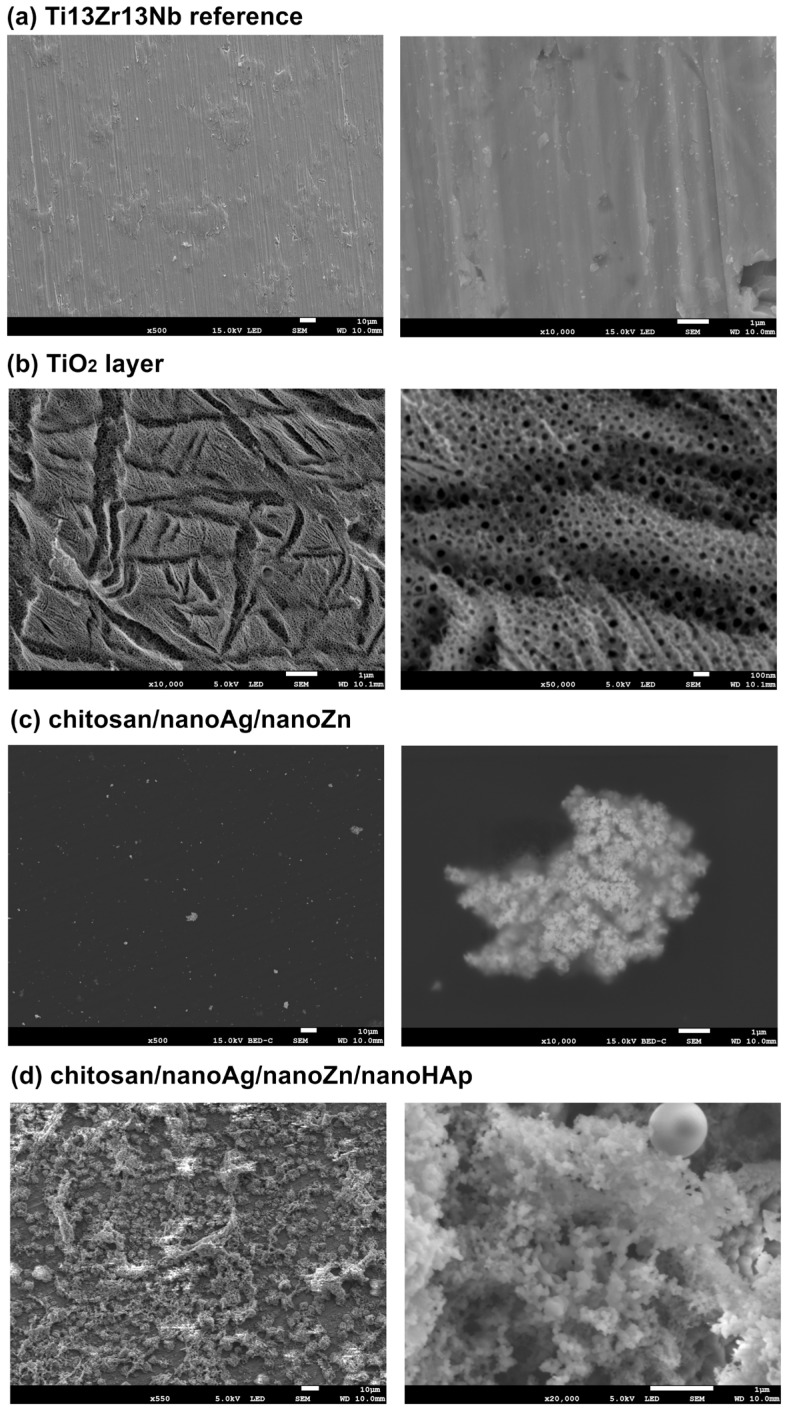
SEM micrographs of the surface microstructure at different magnifications: (**a**) reference Ti13Zr13Nb; (**b**) oxidized TiO_2_ layer; (**c**) chitosan/nanoAg/nanoZn and (**d**) chitosan/nanoAg/nanoZn/nanoHAp composite coating. The pictures are representative of three analyzed specimens for each group.

The morphology of the TiO_2_ nanotube layer is presented in Figure 1. The formed layer consists of nanotubes with an average inner diameter of approximately 36 nm and a wall thickness of about 12 nm. The nanotubes were formed in an irregular manner, with smaller tubes exhibiting inner diameters ranging from 8 nm to 47 nm. For the chitosan-based coatings containing only metallic nanoparticles, the presence of nanoparticle agglomerates can be observed (Figure 1c). The coating is continuous, and no characteristic bubbles resulting from water electrolysis during electrophoretic deposition were detected. In the SEM images of the coating containing nanoHAp (Figure 1d), the distribution of nanohydroxyapatite particles within the chitosan matrix is visible. The particles form agglomerates of various sizes, distributed in a non-uniform manner. The coating exhibits a relatively small thickness, allowing the underlying nanotube layer structure to remain visible.

**Figure 2:**
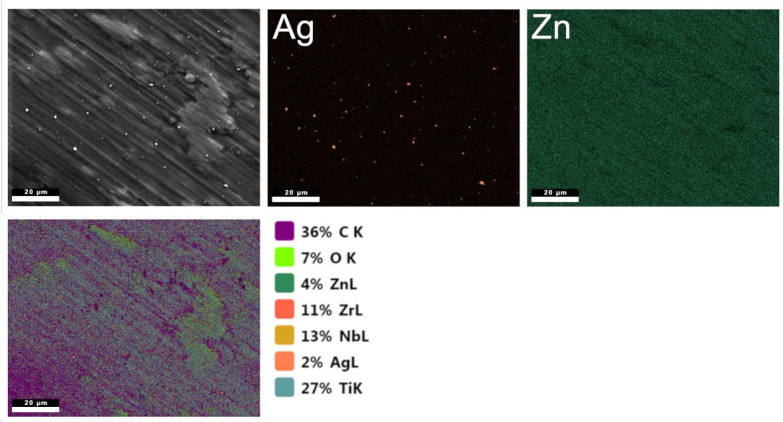
EDS elemental maps and corresponding element identification for the chitosan/nanoAg/nanoZn sample. The pictures are representative of three analyzed specimens for each group.

Figure 2 shows the elemental distribution along with the corresponding percentage composition. A high content of carbon originating from chitosan is visible, as well as a significant amount of oxygen. The map also reveals the distribution of substrate elements. The presence of silver and zinc within the coating was confirmed. Zinc was found to be dissolved in the coating and present in ionic form, whereas silver occurs in the form of nanoparticles.

**Figure 3:**
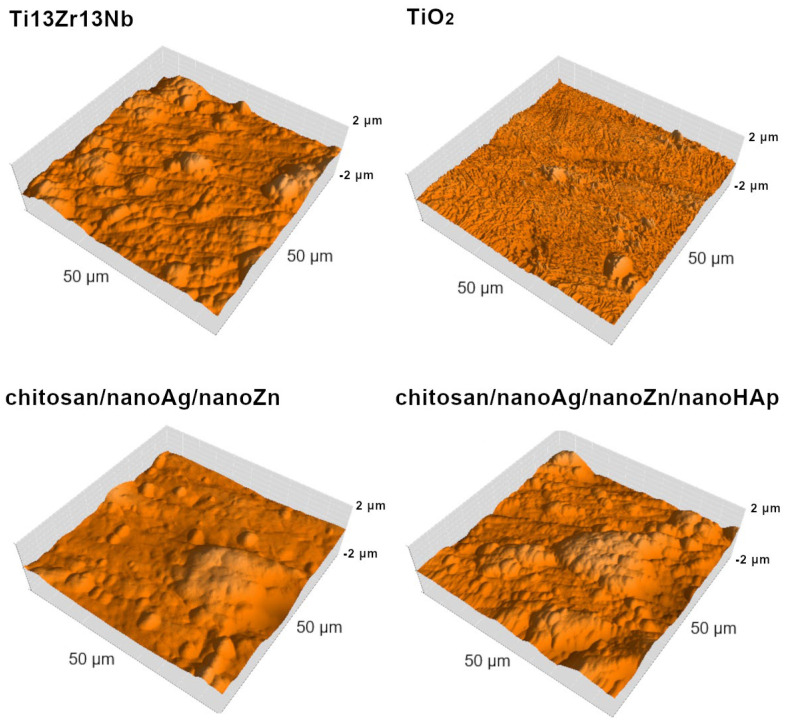
Surface topographies obtained by atomic force microscopy (AFM). The pictures are representative of three analyzed specimens for each group.

**Figure 4:**
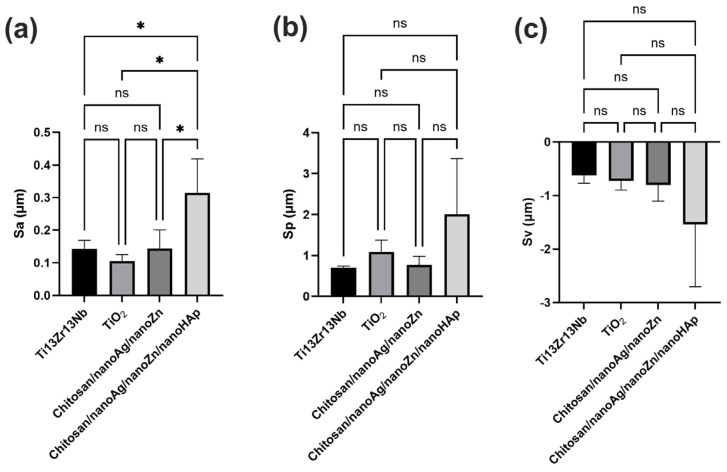
Results of Surface topography parameters Sa (**a**), Sp (**b**) and Sv (**c**). Data are presented as means ± SD from *n* = 3 independent specimens. Significant differences were determined by one-way ANOVA followed by Tukey’s post hoc test. Statistical significance was indicated as follows: * *p* < 0.05 (ns—no statistical difference).

**Figure 5:**
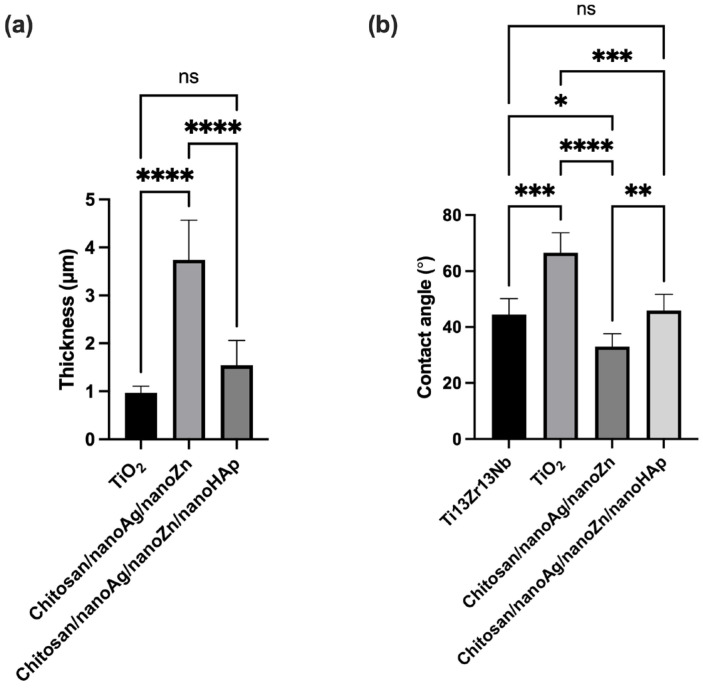
Results of thickness (**a**) and water contact angle (**b**). Data are presented as means ± SD from *n* = 3 independent specimens. Significant differences were determined by one-way ANOVA followed by Tukey’s post hoc test. Statistical significance was indicated as follows: * *p* < 0.05, ** *p* < 0.01, *** *p* < 0.001, **** *p* < 0.0001 (ns—no statistical difference).

Figure 3 presents the AFM surface topography of the investigated materials. Figure 4 and Figure 5 summarize the surface roughness parameters Sa, Sp, and Sv, the thickness of the obtained coatings, and the water contact angle values. Analysis of the Sa parameter, which is used to describe surface roughness, revealed that the chitosan/nanoAg/nanoZn/nanoHAp coatings exhibited the highest roughness, while the TiO_2_ nanotube layer showed the lowest value, reducing the roughness of the reference Ti13Zr13Nb titanium alloy sample. The chitosan/nanoAg/nanoZn coatings displayed a surface roughness very similar to that of the reference alloy. The Sp parameter represents the highest surface peak, whereas Sv indicates the deepest valley. The Sp and Sv values for the reference sample, the nanotube layer, and the TiO_2_/chitosan/nanoAg/nanoZn coating were comparable, indicating similar surface morphology. In contrast, the Sp and Sv parameters for the chitosan/nanoAg/nanoZn/nanoHAp coating differed significantly from those of the reference sample. As is well known, high surface roughness has a positive effect on the biocompatibility and osseointegration of implanted biomaterials [26,27].

The thickness measurements indicated that the obtained coatings were relatively thin, particularly those containing nanohydroxyapatite. The lowest water contact angle was observed for the chitosan/nanoAg/nanoZn coating, while the highest value was recorded for the TiO_2_ layer. The contact angle of the reference Ti13Zr13Nb alloy was comparable to that of the coatings containing nanohydroxyapatite. The fabricated chitosan/nanoAg/nanoZn and chitosan/nanoAg/nanoZn/nanoHAp coatings, as well as the Ti13Zr13Nb alloy, exhibited hydrophilic properties. Low contact angle values, and thus hydrophilicity, may positively influence the biocompatibility of the material. The presence of hydroxyl groups, confirmed by FTIR analysis for both coatings, contributes to their hydrophilic character. The lower contact angle of the chitosan/nanoAg/nanoZn coating may be attributed to its greater thickness. Hydrophilicity of the surfaces is one of the essential factors affecting the first biological response in long-term implant applications. Surfaces with a water contact angle below 90° are generally regarded as hydrophilic, which could favor the interactions with physiological fluids, enhance protein adsorption, and promote cell adhesion-an essential step in processes that initiate osteointegration [28]. Previous investigations have revealed that chitosan-based coatings exhibit pronounced hydrophilicity, which contributes to improved cellular responses and is in favor of biointegration at the implant-tissue interface [28,29].

**Figure 6:**
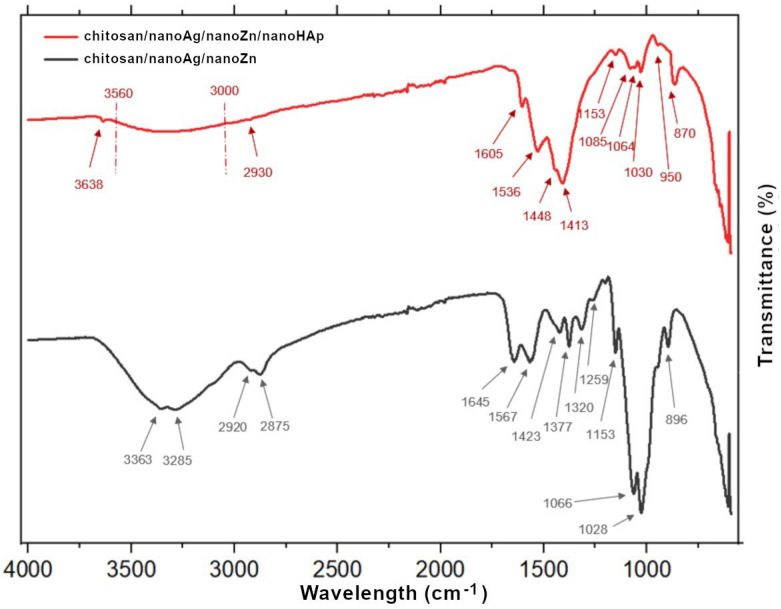
FTIR spectra of chitosan/nanoAg/nanoZn and chitosan/nanoHAp/nanoAg/nanoZn composite coatings.

The results of the FTIR analysis, presented as spectra, are shown in Figure 6 and summarized in Table 3. For the chitosan/nanoAg/nanoZn/nanoHAp coating, the absorption bands in the range of 3285–3363 cm^−1^ correspond to O–H and N–H bonds, as well as to intramolecular hydrogen bonds. The bands at 2920 cm^−1^ and 2875 cm^−1^ correspond to C–H stretching vibrations, which are characteristic of polysaccharides. The presence of N-acetyl groups was confirmed by the bands at 1645 cm^−1^ (C=O of primary amide I), 1567 cm^−1^ (N–H of secondary amide II), and 1320 cm^−1^ (C–N of tertiary amide III). The CH_2_ and CH_3_ groups are confirmed by the bands at 1423 cm^−1^ and 1377 cm^−1^, respectively. The bending vibrations of hydroxyl groups present in chitosan are assigned to the band at 1259 cm^−1^. The absorption band at 1153 cm^−1^ may correspond to the C–O–C bridge, while those at 1066 cm^−1^ and 1028 cm^−1^ are attributed to C–O stretching vibrations. The signal around 896 cm^−1^ corresponds to out-of-plane CH bending in the monosaccharide ring. For the chitosan/nanoAg/nanoZn/nanoHAp coating, the absorption band at 3638 cm^−1^ confirms the presence of hydroxyl groups, while the broad band in the range of 3000–3560 cm^−1^ may be associated with N–H and O–H bonds. A weak band at 2930 cm^−1^ may indicate C–H stretching, characteristic of polysaccharides, though it is less intense than in the chitosan/nanoAg/nanoZn/nanoHAp coating, and the band at 2875 cm^−1^—also associated with C–H stretching—is absent. The presence of N-acetyl groups was confirmed by bands at 1605 cm^−1^ (C=O of amide I) and 1536 cm^−1^ (N–H of amide II).

No absorption band confirming the presence of the C–N bond characteristic of tertiary amide (amide III) was detected, most likely because it overlapped with other bands. The presence of the CO_3_^2−^ functional group was confirmed by the absorption bands at 1148 cm^−1^ and 870 cm^−1^. The CH_2_ and CH_3_ groups are confirmed by the band at 1413 cm^−1^. The absorption band at 1153 cm^−1^ may correspond to the C–O–C bridge, while the bands at 1064 cm^−1^ and 1030 cm^−1^ are attributed to C–O stretching vibrations. The absorption band at 1085 cm^−1^ corresponds to the PO_4_^3−^ group, and the presence of the P–O bond is additionally confirmed by the band at 870 cm^−1^ [30,31,32,33,34,35,36]. The FTIR spectra were interpreted qualitatively to confirm the presence of functional groups originating from chitosan, hydroxyapatite, and metallic additives. In view of the partial overlap of several vibration bands and the thin, heterogeneous nature of the coatings, peak deconvolution and curve fitting were not performed at this stage. This limitation will be addressed in future work through full spectral deconvolution to improve the precision of band assignment.

**Figure 7:**
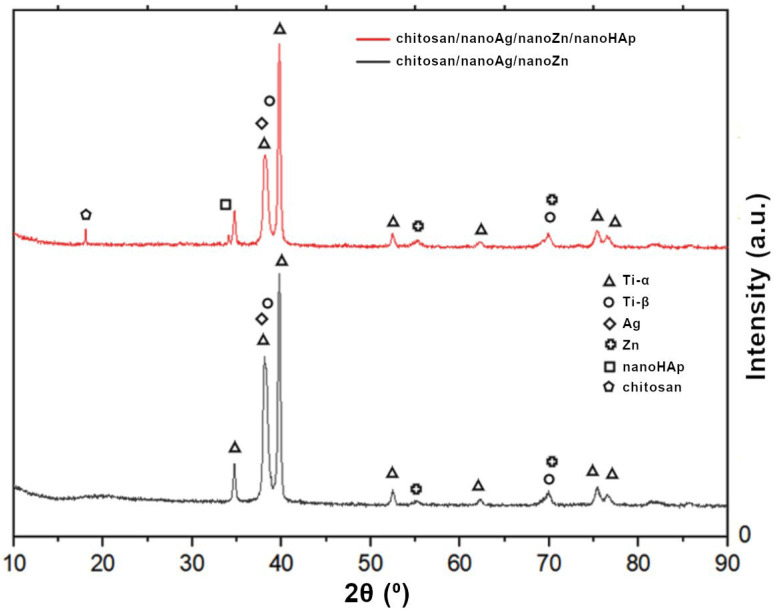
X-ray diffraction (XRD) patterns of chitosan/nanoAg/nanoZn and chitosan/nanoHAp/nanoAg/nanoZn coatings.

The X-ray diffraction (XRD) patterns of the investigated coatings are presented in Figure 7. In both cases, the observed peaks are mainly attributed to the Ti13Zr13Nb substrate. For the/chitosan/nanoAg/nanoZn/nanoHAp sample, the presence of chitosan was identified at 2θ = 18°, and nanohydroxyapatite was also detected at 2θ = 34°. The diffraction peak at 2θ = 38° was assigned to nanosilver, while the peaks at 2θ = 55° and 70° were attributed to zinc. The zinc peak at 2θ = 70° and the silver peak overlapped with the diffraction peaks of the substrate material [31,32,33,34,35,36]. The XRD analysis in this study was intended as a qualitative phase identification. Due to the dominant diffraction signal from the Ti13Zr13Nb substrate and the very low concentration of Ag and Zn nanoparticles (≤0.05 g L^−1^), advanced procedures such as instrumental broadening correction, Rietveld refinement, or texture analysis were not applied. Therefore, the weak reflections attributed to Ag and Zn should be interpreted as indicative.

**Figure 8:**
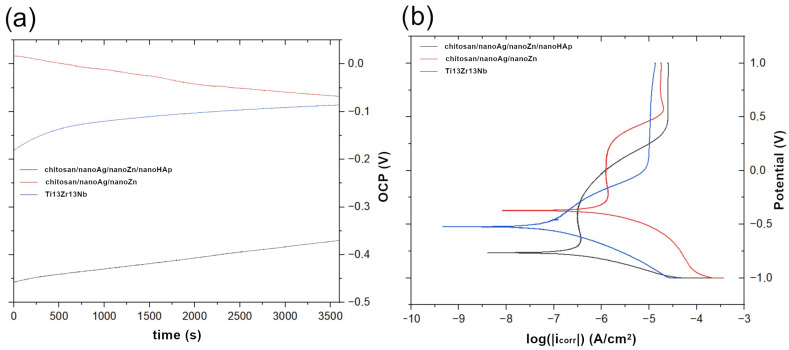
Open-circuit potential (OCP) versus time curves for the reference sample and the obtained coatings (**a**) Potentiodynamic polarization curves for the reference Ti13Zr13Nb sample and the chitosan/nanoAg/nanoZn and chitosan/nanoAg/nanoZn/nanoHAp coatings (**b**). The pictures are representative of three analyzed specimens for each group.

**Figure 9:**
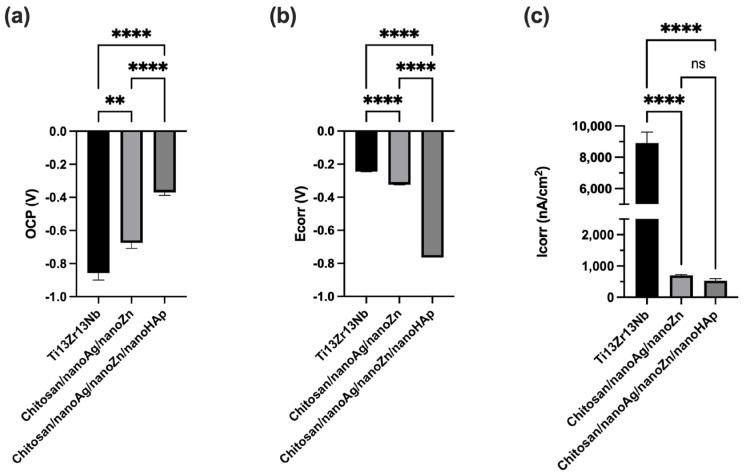
Results of open-circuit potential (**a**), corrosion potential (**b**), and corrosion current density (**c**) for the reference sample and the obtained chi-tosan/nanoAg/nanoZn and chitosan/nanoAg/nanoZn/nanoHAp coatings. Data are presented as means ± SD from *n* = 3 independent specimens. Significant differences were determined by one-way ANOVA followed by Tukey’s post hoc test. Statistical significance was indicated as follows: ** *p* < 0.01, **** *p* < 0.0001 (ns—no statistical difference).

Figure 8 presents the open-circuit potential (OCP) curves recorded for the reference sample and the fabricated coatings (a) and the potentiodynamic polarization curves for the reference Ti13Zr13Nb alloy and the chitosan-based coatings (b). All samples exhibited similar curve profiles and demonstrated passivation behavior. Figure 9 summarizes the corrosion current density, corrosion potential, and open-circuit potential determined by extrapolation of the Tafel plots. The most negative corrosion potential was recorded for the chitosan/nanoAg/nanoZn/nanoHAp coating (−0.764 V). The reference Ti13Zr13Nb alloy exhibited the most positive corrosion potential (−0.245 V), while an intermediate value was obtained for the chitosan/nanoAg/nanoZn coating. For both coatings, the corrosion potential shifted toward more negative values compared to the Ti13Zr13Nb substrate. The best corrosion resistance was observed for the chitosan/nanoAg/nanoZn/nanoHAp coatings, which showed the lowest corrosion current density (529.55 nA/cm^2^). A higher, though still lower than that of the uncoated titanium alloy (8907.64 nA/cm^2^), corrosion current density was measured for the chitosan/nanoAg/nanoZn coatings (699.57 nA/cm^2^). These results indicate that the applied coatings enhanced the corrosion resistance of the Ti13Zr13Nb alloy. The open-circuit potential values differed significantly from the corrosion potential of the obtained coatings. For the chitosan/nanoAg/nanoZn coating, the OCP shifted markedly toward negative values, while for the chitosan/nanoAg/nanoZn/nanoHAp coating it shifted toward positive values. The deterioration in corrosion resistance of the coating without nanoHAp may be attributed to the higher content of silver nanoparticles forming agglomerates. Such inhomogeneities, caused by the presence of numerous silver agglomerates, may contribute to a decrease in corrosion resistance [37]. The open-circuit potential of the reference sample was also shifted toward negative values, reaching −0.857 V.

**Figure 10:**
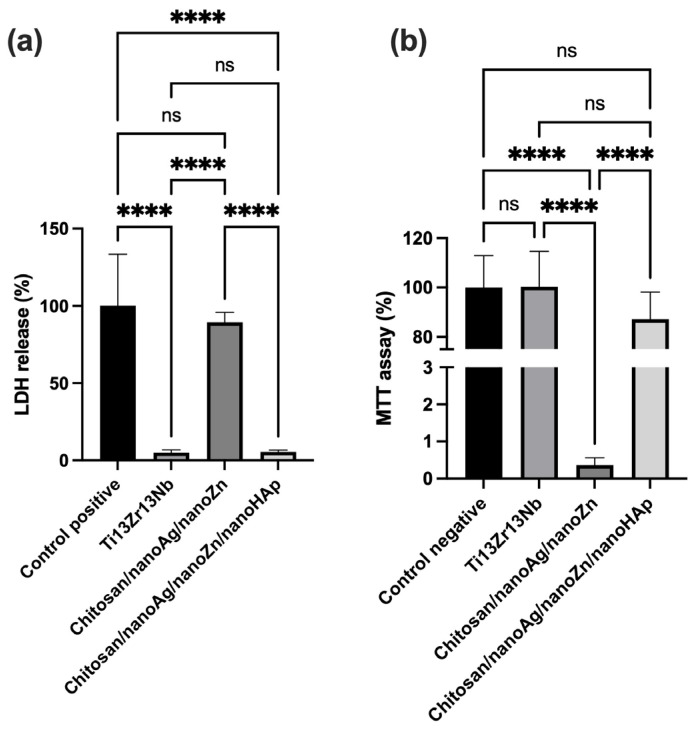
Results of LDH (**a**) and MTT (**b**) assays under indirect-contact conditions for the investigated samples. Data are presented as means ± SD from *n* = 3 independent specimens. Significant differences were determined by one-way ANOVA followed by Tukey’s post hoc test. Statistical significance was indicated as follows: **** *p* < 0.0001 (ns—no statistical difference).

The results of the cytotoxicity tests (MTT and LDH) conducted in indirect contact with human osteoblast cells are presented in Figure 10. In the MTT assay, cell viability was determined relative to the negative control (viable cells), whereas in the LDH assay it was evaluated relative to the positive control (dead cells). The results indicate high biocompatibility and cell survival, exceeding 85%, for the chitosan/nanoAg/nanoZn/nanoHAp coatings. However, very low cell viability—close to zero—was observed for the chitosan/nanoAg/nanoZn coatings, indicating high cytotoxicity. The cell viability for these coatings was only 0.364%. The extremely low cell viability observed for the chitosan/nanoAg/nanoZn coatings may result from an excessive amount of metallic nanoparticles incorporated into the coating, which could have been released uncontrollably in large quantities, leading to cell death. It is also possible that the addition of nanohydroxyapatite affected nanoparticle migration during the electrophoretic deposition process, thereby reducing their concentration within the coating. Moreover, the presence of nanohydroxyapatite itself may have enhanced the biocompatibility of the coatings, as confirmed by the obtained results. The LDH test supported these findings: LDH release reached 89.48% for the chitosan/nanoAg/nanoZn coatings, 5.04% for the Ti13Zr13Nb alloy, and 5.40% for the coatings containing nanohydroxyapatite. The Ti13Zr13Nb alloy exhibited high biocompatibility with human cells, as confirmed by the experimental results.

These findings are in accordance with previous work that has demonstrated that excessive Ag^+^ and Zn^2+^ ion release can result in the induction of oxidative stress, mitochondrial dysfunction, and apoptotic pathways in osteoblasts, thereby reducing cell viability [38,39,40,41]. Conversely, the presence of hydroxyapatite nanoparticles enhances biocompatibility through protein adsorption, which favors the adhesion and proliferation of osteoblasts [42,43]. Hydroxyapatite would provide calcium and phosphate ions, which are highly valuable for cell differentiation and bone tissue regeneration, counterbalancing the probable cytotoxic effect induced by metallic nanoparticles. The biocompatibility of the Ti13Zr13Nb alloy, as previously deduced from both tested assays, is in agreement with literature outcomes highlighting low cytotoxicity and excellent behavior as a new generation of β-type titanium alloys for biomedical applications [44,45]. It can therefore be inferred that the addition of nanohydroxyapatite in the chitosan matrix serves not only to stabilize the nanoparticle distribution during electrophoretic deposition but also to improve the biological behavior of the coatings by means of ion release reduction and increased osteoblast viability. Studies on chitosan-based systems incorporating silver and/or zinc have thus far demonstrated that both Ag^+^ and Zn^2+^ exhibit strongly concentration dependent and often inconsistent cytotoxicity thresholds. For example, Peng et al. demonstrated that human HS27 dermal fibroblasts exposed to chitosan coated AgNPs showed a sharp decrease in viability at approximately 30 µg/mL after 24 h [46], but Putthanuparp et al. [47] determined that human gingival fibroblasts exhibited >70% viability following as long as 24 h exposure to 10 µg/mL AgNPs, indicating a significantly lower apparent toxicity threshold in that model. Similar broad variability has been reported for zinc-containing systems. Cierech et al. [48] showed that HeLa cells did not exhibit any significant loss of viability up to 20 mg L^−1^ (≈20 µg/mL) ZnO NPs after 24 h, while at 30 mg L^−1^ viability was reduced to ~61% (±5.1%). In contrast, Han et al. [49] demonstrated that macrophages tolerated Zn^2+^ concentrations of 4.7 and 37.5 µM without significant cytotoxicity but displayed a gradual viability decline at concentrations ≥ 300 µM, with an IC_50_ of approximately 510 µM. More recently, Maheo et al. [50] reported an IC_50_ of 62.6 µg/mL for chitosan assisted ZnO nanoparticles in MG63 osteoblast like cells following 24 h exposure; however, Kazimierczak et al. [51] demonstrated that even modest increases in Zn content within chitosan–agar–hydroxyapatite scaffolds triggered significant cytotoxicity due to the burst release of Zn^2+^ during the first 24 h. Collectively, these studies unequivocally demonstrate that the literature reports scattered and sometimes apparently contradictory cytotoxicity thresholds for Ag^+^ and Zn^2+^, emphasizing the importance of caution when defining “safe” ion concentrations in multifunctional chitosan-based systems.

## 4. Conclusions

In this study, biopolymer composite coatings based on chitosan containing silver, zinc, and hydroxyapatite nanoparticles were successfully fabricated on Ti13Zr13Nb alloy substrates using the electrophoretic deposition (EPD) technique. The structural, electrochemical, and biological analyses demonstrated that both surface pretreatment and the incorporation of nanoparticles significantly affected the coating properties. The produced coatings were continuous, uniform, and exhibited the presence of characteristic functional groups confirmed by FTIR and phase composition verified by XRD. AFM and SEM analyses revealed that the surface morphology and roughness were suitable for enhancing cell adhesion. Electrochemical tests in simulated body fluid (SBF) showed that the chitosan/nanoAg/nanoZn/nanoHAp coatings exhibited the highest corrosion resistance, with corrosion current density values substantially lower than that of the uncoated Ti13Zr13Nb alloy. Contact angle measurements confirmed that all coatings were hydrophilic, which is favorable for improving cell–material interactions. Biological tests using the hFOB 1.19 osteoblast cell line confirmed excellent biocompatibility of the coatings containing hydroxyapatite (>85% cell viability), whereas coatings without HAp exhibited increased cytotoxicity, likely due to excessive release of metallic nanoparticles.

Overall, the incorporation of nanohydroxyapatite into chitosan-based coatings not only improved their corrosion resistance but also enhanced their cytocompatibility, making the developed Ti13Zr13Nb/chitosan/nanoAg/nanoZn/nanoHAp system a promising candidate for biomedical applications, particularly as a functional coating for titanium-based implants.

## Figures and Tables

**Table 1:** The chemical composition of the Ti13Zr13Nb alloy wt.%.

Element	Nb	Zr	Fe	C	N	O	Ti
wt.%	13.0	13.0	0.05	0.04	0.019	0.11	remainder

**Table 2:** Details of specimens description and EPD parameters.

Specimens Description	Electrochemical Oxidation	Coating Deposition Parameters
Ti13Zr13Nb	-	-
TiO_2_	H_3_PO_4_ + HF; 20 V; 20 min	-
chitosan/nanoAg/nanoZn		1 g/L chitosan + 0.05 g/L AgNPs + 0.05 g/L ZnNPs; 20 V; 1 min
chitosan/nanoAg/nanoZn/nanoHAp		1 g/L chitosan + 0.05 g/L AgNPs + 0.05 g/L ZnNPs + 2.5 g/L HApNPs; 20 V; 1 min

**Table 3:** FTIR wavenumbers and corresponding chemical bonds/functional groups identified for tested coatings.

Chitosan/nanoHAp/nanoAg/nanoZn		Chitosan/nanoAg/nanoZn	
Wavenumber (cm^−1^)	Bond/Functional Group	Wavenumber (cm^−1^)	Bond/Functional Group
3638	–O–H	3363	–O–H/N–H
3560–3000	–O–H/N–H	3285	–O–H/N–H
2930	C–H	2920	C–H
1605	C=O	2875	C–H
1536	N–H	1645	C=O
1448	CO_3_^2−^	1567	N-H
1413	CH_2_/CH_3_	1423	CH_2_/CH_3_
1153	C–O–C	1377	CH_2_/CH_3_
1085	PO_4_^3−^	1320	C–N
1064	C–O	1259	–O–H
1030	C–O	1153	C–O–C
950	P–O	1066	C–O
870	CO_3_^2−^	1028	C–O
-	-	896	C-H

## Data Availability

The raw data supporting the conclusions of this article will be made available by the authors on request.
